# Supplementary material for: Genome-wide identification, characterization and gene expression of BES1 transcription factor family in grapevine (Vitis vinifera L.)
Source: Sci Rep. 2023 Jan 5;13:240. doi: 10.1038/s41598-022-24407-y (PMC9816167; doi:10.1038/s41598-022-24407-y)
Supplement: Supplementary file 3 — Supplementary Information. [file 41598_2022_24407_MOESM3_ESM.zip › Vvi_Atr/Vitis_vinifera.PN40024.v4.dna_sm.toplevel.fa.vs.Amborella_trichopoda.AMTR1.0.dna_sm.toplevel.fa.html/Atr-AmTr_v1.0_scaffold00163.html]

|  |  |  |  |  |  |  |  |  |  |  |  |  |  |
| --- | --- | --- | --- | --- | --- | --- | --- | --- | --- | --- | --- | --- | --- |
| Duplication depth | Reference chromosome | Collinear blocks | | | | | | | | | | | |
| 0 | Atr-ERN09043 |  |  |  |  |  |  |  |  |  |  |  |  |
| 0 | Atr-ERN09044 |  |  |  |  |  |  |  |  |  |  |  |  |
| 0 | Atr-ERN09045 |  |  |  |  |  |  |  |  |  |  |  |  |
| 0 | Atr-ERN09046 |  |  |  |  |  |  |  |  |  |  |  |  |
| 0 | Atr-ERN09047 |  |  |  |  |  |  |  |  |  |  |  |  |
| 0 | Atr-ERN09048 |  |  |  |  |  |  |  |  |  |  |  |  |
| 0 | Atr-ERN09049 |  |  |  |  |  |  |  |  |  |  |  |  |
| 1 | Atr-ERN09050 |  | Vvi-Vitvi04g01176\_t002 |  |  |  |  |  |  |  |  |  |  |
| 1 | Atr-ERN09051 |  | \| \| |  |  |  |  |  |  |  |  |  |  |
| 1 | Atr-ERN09052 |  | \| \| |  |  |  |  |  |  |  |  |  |  |
| 1 | Atr-ERN09053 |  | \| \| |  |  |  |  |  |  |  |  |  |  |
| 1 | Atr-ERN09054 |  | \| \| |  |  |  |  |  |  |  |  |  |  |
| 1 | Atr-ERN09055 |  | \| \| |  |  |  |  |  |  |  |  |  |  |
| 1 | Atr-ERN09056 |  | Vvi-Vitvi04g01184\_t002 |  |  |  |  |  |  |  |  |  |  |
| 1 | Atr-ERN09057 |  | \| \| |  |  |  |  |  |  |  |  |  |  |
| 1 | Atr-ERN09058 |  | \| \| |  |  |  |  |  |  |  |  |  |  |
| 1 | Atr-ERN09059 |  | Vvi-Vitvi04g01185\_t001 |  |  |  |  |  |  |  |  |  |  |
| 1 | Atr-ERN09060 |  | \| \| |  |  |  |  |  |  |  |  |  |  |
| 1 | Atr-ERN09061 |  | Vvi-Vitvi04g01189\_t002 |  |  |  |  |  |  |  |  |  |  |
| 1 | Atr-ERN09062 |  | \| \| |  |  |  |  |  |  |  |  |  |  |
| 1 | Atr-ERN09063 |  | \| \| |  |  |  |  |  |  |  |  |  |  |
| 1 | Atr-ERN09064 |  | \| \| |  |  |  |  |  |  |  |  |  |  |
| 1 | Atr-ERN09065 |  | \| \| |  |  |  |  |  |  |  |  |  |  |
| 1 | Atr-ERN09066 |  | Vvi-Vitvi04g01190\_t001 |  |  |  |  |  |  |  |  |  |  |
| 1 | Atr-ERN09067 |  | \| \| |  |  |  |  |  |  |  |  |  |  |
| 1 | Atr-ERN09068 |  | Vvi-Vitvi04g01193\_t002 |  |  |  |  |  |  |  |  |  |  |
| 0 | Atr-ERN09069 |  |  |  |  |  |  |  |  |  |  |  |  |
| 0 | Atr-ERN09070 |  |  |  |  |  |  |  |  |  |  |  |  |
| 0 | Atr-ERN09071 |  |  |  |  |  |  |  |  |  |  |  |  |
| 0 | Atr-ERN09072 |  |  |  |  |  |  |  |  |  |  |  |  |
| 0 | Atr-ERN09073 |  |  |  |  |  |  |  |  |  |  |  |  |
| 0 | Atr-ERN09074 |  |  |  |  |  |  |  |  |  |  |  |  |
| 0 | Atr-ERN09075 |  |  |  |  |  |  |  |  |  |  |  |  |
